# Injection of Hydrogen Peroxide into the Peritoneal Cavity

**Published:** 1893-11-04

**Authors:** Benjamin Ward Richardson


### Injection of Hydrogen Peroxide into the Peritoneal Cavity.

By Sir Benjamin Ward Richardson, M.D., F.R.S.

Dr. Frederick H. Wiggin, of New York, has published in the *Medical Record* for October 7th, a notice of two cases in which Peroxide of Hydrogen Solution was introduced into the open peritoneal cavity with good results. In the first case during the course of an operation for removal of a purulent ovarian cyst, parts of the contents escaped into the peritoneal cavity. Half-a-pound of hydrogen dioxide solution of full medicinal strength, 10 or 15 volumes, was poured into the cavity, and the patient made a prompt and uneventful recovery.

In the second case a boy of sixteen was being submitted to resection of the small intestine by Manusell's method. There was an escape of fæcal matter into the peritoneal cavity, and at once a liberal quantity of peroxide solution (amount not stated) was introduced into the cavity. General peritonitis and fibrinous exudation was present at the time, but the peritonitis subsided, the temperature never rose as high after the operation as previous to it, and the boy up to date of report, three weeks it would seem after the operation, had made a good recovery.

As, therapeutically, peroxide of hydrogen is one of my children, I am much gratified to hear of so good a result from its use in the cases named, and I congratulate Dr. Wiggin on his success. At the same time, lest the value of the peroxide should be impaired by a divergence from the course pursued by Dr. Wiggin, I wish specially to point out that he used the remedy in cases where the peritoneal cavity was at the time open. It might be inferred that because the practice succeeded under those circumstances, therefore the fluid might be injected into the closed peritoneum when there is purulent fluid in it, or other organic matter which the peroxide might destroy. I have given this subject considerable attention, and have made careful experiment upon it, as may be seen in "Asclepiads" xxix. page 11, and xxxi., pages 211-13. In one of these experimental researches I endeavoured to restart the circulation in an animal dead from the inhalation of chloroform by injecting the solution of the peroxide into the peritoneun. I thought by this plan I should get direct absorption of oxygen into the great veins, and in this manner feed the lung from the right side with oxygenated blood. I thought also that by this same means I should establish by a *vis a tergo* a current onwards over the lung into the left heart, and, lastly, I thought I might excite the diaphragm into renewed action and so set up a new kind of artificial respiration. The injection of an ounce of the fluid through the hypodermic needle into the peritoneum of the dead animal (a cat) led to the most startling effects. There was no return of consciousness, and there did not appear to be any return of the circulation, but the breathing which had ceased for several minutes was restored most perfectly. It recommenced and proceeded in regular rhythm, and continued for fully five minutes, then it gradually ceased, and on introduction of more solution did not respond with any vigour, although a few feeble movements were perceptible. It was clear that the irritability of the diaphragm and other muscles of respiration had been played out, and that no further reanimation could be expected. When my mind was quite made up on this point I conducted a post-mortem examination of the body of the animal, and then discovered readily enough what had occurred. There had been, as I had anticipated, very complete absorption of oxygen, for some remaining fluid in the cavity contained no oxygen, none could be set free by manganese or other liberating agent, and the liver and all the surrounding tissues were of bright red colour as if the parts had been dipped into and retained in oxygen gas. The heart was perfectly dead, responding to no stimulus, But here was the curious part: the heart and the large veins leading to it and the pulmonary artery were all charged with bright red oxygenated blood, containing free oxygen. Some of this blood, collected in a watch glass, contained bubbles of the gas, which relighted a small match not quite extinguished. I doubt, in fact, whether there had been any degree of absorption of oxygen of any kind into the corpuscles, for when the blood was set aside all the oxygen was evolved from it, and ordinary venous blood remained.

Blood thus charged with a free gas could not circulate through the minute vessels. It would bring the circulation to a standstill, as in the experiment of blowing air into a vein, and from which we see that the lungs are not merely necessary as the receivers of oxygen from the air, but by their infinite ramifications are the means by which, through surface and exposure to the oxygen of the air in the capillaries of the general circuit, there is absorption of oxygen into the red particles of the blood.

The practical upshot of these observations is at this moment important, it may be, critical. I have no idea of danger from injection of peroxide solution into any open cavity. I see no objection as to its injection into the cellular tissue, which it would simply inflate as common air inflates, without risk of too rapid an introduction into the blood. I see no objection in blowing the gas in small quantities into cavities in the lung, nor even into the cellular structure of the lung. I have suggested these methods, and have explained that the diffusion of oxygen through the tissue of the lungs would arterialize the blood in the lung in asphyxia, and that the mechanical pressure exerted within the pleura against the elastic lung structure could be made, through the up and down movement of the piston of the injecting syringe, to compress the arterial blood onwards towards the left heart, and to bring up venous blood to the right side, so as to establish what I called many years ago *artificial circulation*. In these directions there is a very wide extension for the employment of the peroxide, and I feel that Dr. Wiggin's new extension has quite a remarkable bearing as to therapeutic use. Let it only be remembered that the injected fluid be not permitted to enter rapidly into the direct blood current, nor be so used that it shall explode by too rapid an escape in such cavities as the middle ear or the cranium, and the only two dangers from it that I am at this moment cognizant of are off the field.

